# The Effect of Implicit Preferences on Food Consumption: Moderating Role of Ego Depletion and Impulsivity

**DOI:** 10.3389/fpsyg.2016.01699

**Published:** 2016-11-09

**Authors:** Yan Wang, Jinglei Zhu, Yi Hu, Yuan Fang, Guosen Wang, Xianghua Cui, Lei Wang

**Affiliations:** ^1^School of Psychology and Cognitive Science, East China Normal UniversityShanghai, China; ^2^Management of Technology and Education Department, The second Affiliated Hospital of Kunming Medical UniversityKunming, China; ^3^The Psychological Counseling Room, Shanghai Wanping Middle SchoolShanghai, China

**Keywords:** implicit preferences, ego depletion, trait impulsivity, stop signal task, food consumption

## Abstract

Ego depletion has been found to moderate the effect of implicit preferences on food consumption, such that implicit preferences predict consumption only under a depleted state. The present study tested how trait impulsivity impacts the effect of implicit preferences on food consumption in a depleted condition. Trait impulsivity was measured by means of self-report and a stop signal task. Results showed that both self-reported impulsivity and behavioral impulsivity moderated the ‘depletion and then eating according to implicit preferences’ effect, albeit in different ways. Participants high in self-reported impulsivity and low in behavioral impulsivity were more vulnerable to the effect of depletion on eating. The implications of these results for extant theories are discussed. Future research is needed to verify whether or not trait impulsivity is associated with vulnerability to depletion across different self-control domains.

## Introduction

The abundance of high-calorie foods in modern society causes some people to consume in excess of their long-term health or weight-management goals (Novak and Brownell, 2011). However, not everyone overeats under the same conditions. Some may find the temptation to eat difficult to resist and become unable to control their impulses under certain circumstances. The dual-systems perspective of impulse and self-control provides a framework for understanding the determinants of health-related behaviors (e.g., eating, drinking, drug abuse; Hofmann et al., 2009b). This perspective draws from the reflective-impulsive model (RIM; Strack and Deutsch, 2004), which suggests that behaviors are determined by the interplay between the impulsive and reflective systems, to multiple domains of self-control (Hofmann et al., 2008). The impulsive system consists of associative clusters reflecting an organism’s learning history. This system drives behavior through an automatic appraisal of a stimulus’s affective and motivational properties. In contrast, the reflective system guides behavior through personal standards, reasoned evaluations of pros and cons, and long-term goals. Furthermore, certain situational and dispositional boundary conditions, or moderators, may shift the relative impact of impulsive and reflective precursors on behavior (Hofmann et al., 2009b). The present research conjoins and examines the interaction between situational differences in ego depletion and dispositional differences in impulsivity.

Food consumption quantity is largely determined by the availability of food and by its appeal (Haynes et al., 2015). Not everyone likes calorie-rich foods to the same degree (Hofmann et al., 2011). Therefore, individual differences in impulse strength toward specific foods should be considered in the study of the human diet (Friese et al., 2008b). According to the RIM, the impulsive system comprises an associative network and activates behavioral schemas through the spreading of activation and the automatic triggering of impulses (Strack and Deutsch, 2004). A valid impulse measure should apply to a specific stimulus of interest, and be sensitive to individual differences in impulse strength (Hofmann et al., 2009b). These criteria permit the use of implicit measures, such as the implicit association test (IAT, Greenwald et al., 1998) and the affect misattribution procedure (AMP, Payne et al., 2005), for indexing impulses. The extent to which implicit measures predict eating behavior depends on multiple situational (e.g., ego depletion) and dispositional moderators (e.g., trait self-control). These moderators are consistent with the dual-systems perspective (Friese et al., 2008a).

According to the strength model of self-control (Baumeister and Heatherton, 1996), exerting self-control quickly consumes a domain general resource and leads to a state of reduced self-regulatory resources called ego depletion (Baumeister et al., 1998). An everyday experience sampling study found that people are more vulnerable to impulses after cumulative depletion (Hofmann K. et al., 2012). Some studies have employed unhealthy food consumption (e.g., ice cream) during a bogus food taste tests as the dependent task, revealing that depleted individuals eat more than those who were not depleted (Baumeister et al., 2005; Inzlicht and Kang, 2010; Imhoff et al., 2014). However, several studies have found no significant main effects of ego depletion in the sense of eating after depletion (Dingemans et al., 2009; Stillman et al., 2009). A possible explanation for this discrepancy is variance in impulse variation. The main effect of ego depletion on consumption only emerges in a given sample of individuals who have higher mean impulses toward specific food (Friese and Hofmann, 2009). Thus, measuring impulses can help to reconcile the inconsistent results concerning the effect of ego depletion on eating.

Studies have investigated the relationship between ego depletion and impulsive precursors in predicting eating. Two studies measured automatic food attitudes with the Single Category IAT (SC-IAT), demonstrating that this instrument predicted food consumption (i.e., candy and potato crisps) for depleted participants but not for non-depleted participants (Hofmann et al., 2007; Friese et al., 2008b). These studies directly support the moderating role of ego depletion in impulsive eating. Therefore, existing evidence supports the view that eating behavior is more impulsively than reflectively driven when ego depleted.

Some trait and individual difference variables may moderate the ego depletion effect (Hagger et al., 2010). Yet very little understanding of potential moderators exists (Lurquin et al., 2016). According to capacity-based theories, self-control is a dispositional, trait-like construct that differs across individuals (e.g., Tangney et al., 2004; de Ridder et al., 2012). According to this view, some people have higher overall self-control capacity and are less vulnerable to the ego depletion effect (Baumeister et al., 2006). Accordingly, trait self-control and trait impulsivity differences potentially moderate the ego depletion effect (Duckworth and Kern, 2011).

Trait self-control is defined as the ability to refrain from acting on one’s inner impulses (Tangney et al., 2004). Evidence suggests that individuals high in trait self-control are less vulnerable to the effect of ego depletion, as reflected in subsequent self-control outcomes such as aggression (DeWall et al., 2007) and alcohol consumption (Muraven et al., 2005). Studies testing the interaction between trait self-control and ego depletion in the domain of eating behavior have been inconclusive. One study found that participants high in trait self-control were more vulnerable to depletion (Imhoff et al., 2014). However, another study suggested that the effect of ego depletion on eating behavior was independent of trait self-control (Hagger et al., 2013). In a third study, high trait self-control was found to attenuate the effect of ego depletion on eating after accounting for implicit preferences toward specific food (Wang et al., 2015).

Trait impulsivity is defined as a chronic and general tendency toward quick, unplanned reactions to stimuli without considering the consequences (Jasinska et al., 2012). Impulsivity is a multidimensional construct (Whiteside and Lynam, 2001) and has been investigated by self-report personality questionnaires and laboratory behavioral tasks (Reynolds et al., 2006). Self-report questionnaires, such as the Eysenck Impulsiveness Questionnaire (Eysenck et al., 1985) and Barratt Impulsiveness Scale (Patton et al., 1995), define impulsivity as an inability to inhibit inappropriate behaviors, wait or act with forethought. These questionnaires ask participants to rate themselves on items like “I do things without thinking” and “I have trouble controlling my impulses.” Behavioral tasks measure overt behavior related to specific impulsivity dimensions. The stop signal task is known as a measure of response inhibition, and impulsivity is defined as an inability to inhibit a motor response in a laboratory setting in such tasks (Logan et al., 1997). Self-report and behavioral tasks have been found to assess different aspects of impulsive behavior (Stahl et al., 2014). However, both measures are linked to increased food intake (Guerrieri et al., 2007a,b; Jasinska et al., 2012). For example, self-reported impulsivity and inefficient response inhibition are positively associated with unhealthy eating (Jasinska et al., 2012). A longitudinal study found that implicit snack food preferences interact with response inhibition in predicting weight gain after 1 year. Participants with strong implicit preferences for snack foods and less effective response inhibition gained the most weight (Nederkoorn et al., 2010). However, no known study has directly tested the moderating effect of trait impulsivity on ego depletion.

The current study investigated the interaction between ego depletion and trait impulsivity and their moderation of implicit preferences on eating. Based on previous findings (Hofmann et al., 2007; Friese et al., 2008b), we predicted a two-way interaction between ego depletion and implicit preferences. We sought to extend existing literature by examining the moderating role of trait impulsivity in the ‘depletion and then eating according to implicit preferences’ effect. Due to multidimensionality of the impulsivity construct (Whiteside and Lynam, 2001), we examined both self-report and behavioral impulsivity measures (i.e., response inhibition). Self-reported impulsivity and trait self-control were thought to represent two end points of the same dimension (Duckworth and Kern, 2011; de Ridder et al., 2012). Therefore, low self-reported impulsivity might interact with ego depletion in a similar way as trait self-control (Muraven et al., 2005; DeWall et al., 2007; Wang et al., 2015). On the other hand, response inhibition is required for successful self-control (Hofmann W. et al., 2012; Inzlicht et al., 2014). The majority of tasks employed in ego depletion studies involve the inhibition of prepotent responses (Baumeister, 2014). Moreover, individuals low in response inhibition (i.e., high in behavioral impulsivity) are more strongly influenced by implicit preferences (Houben and Wiers, 2009; Hofmann et al., 2009a). Therefore, low behavioral impulsivity may serve as a buffer against the effect of depletion on subsequent impulsive eating. We predicted that trait impulsivity, ego depletion, and implicit preferences would interact to influence food consumption. Specifically, the effect of implicit preferences on food consumption under a depleted condition existed only in individuals with high trait impulsivity.

## Materials and Methods

We report how we determined our sample size, all data exclusions, all manipulations, and all measures used (Simmons et al., 2011).

### Participants

Participants were 100 female undergraduate students from a Chinese university. The sample size was determined in advance to be comparable with previous ego depletion studies using food consumption as the outcome variable (e.g., Hofmann et al., 2007, 2009a; Hagger et al., 2013). We limited our sample to young females, because food cravings are more prevalent in females than in males (Weingarten and Elston, 1991). Food cravings also decrease with age (Pelchat, 1997). Thus, food consumption may pose a greater threat to young females’ self-control. The sample received course credit or monetary compensation (∼US$5) for participating. The mean age of the sample was 21.3 years (*SD* = 2.4 years) and the mean BMI was 20.7 (*SD* = 2.1). Data from five subjects were excluded because of eating more than 2.5 standard deviations from the overall mean (*n* = 2; McClelland, 2000), previously participating in studies where they were offered food (*n* = 2; Lawrence et al., 2015), or being a multivariate outlier in the multiple regression analyses (*n* = 1; Studentized deleted residual >3 and Cook’s Distance >0.15; Friese et al., 2015). The inclusion of the five excluded participants in the analyses did not change the results. Participants were randomly assigned to one of two conditions: No-depletion (*n* = 47) and depletion (*n* = 48).

### Procedure

All participants took part in the experiment individually in two sessions. The first session took place between 10:30 and 11:30 am or 3:30 and 5:30 pm, to ensure hunger would be similar across participants. Each participant was briefed by a female experimenter. Participants first completed a perception task, which actually measured implicit preferences for chocolate. Next, participants performed a task that required them to cross out letters on two pages of text, which manipulated their depletion level. Participants then tasted and rated several plates of Hershey chocolates during the taste test phase. Finally, participants indicated when and what they last ate before the experiment, what they thought was the purpose of the study, and their personal information (i.e., age, height, and weight).

The second session took place about 2 weeks later. Each participant first performed the behavioral impulsivity task on a computer. Participants then completed the self-reported trait impulsivity questionnaire.

Participants were debriefed via email after data collection had been completed. The University Committee on Human Research Protection of East China Normal University approved this study.

### Ego Depletion Manipulation

We used an established “e-crossing” task to manipulate ego depletion (Baumeister et al., 1998). Participants were given two pages of paper with printed text. On the first page, participants were asked to cross out all the letters “e”. The second page contained different instructions for each condition. Participants in the no-depletion condition were asked to cross out all the letters “e”. Participants in the depletion condition were required to change their behavior by following new rules. Specifically, they had to cross out all the letters “e” unless followed by a vowel or preceded by a vowel two letters before. Thus, the depletion condition required participants to override the habitual response formed during the first page.

Participants completed a 20-item Positive and Negative Affect Schedule (Watson et al., 1988) immediately after the task, which measured their current mood on a 5-point rating scale. As a manipulation check, participants were asked to rate on a 6-point scale how effortful it was to follow the instructions for crossing out letters.

### Measures

#### Implicit Measure

Implicit preferences toward chocolate were measured with a personalized Single Category IAT (SC-IAT, Karpinski and Steinman, 2006). The chocolate SC-IAT consists of two stages. Participants completed these stages in the same order (Karpinski and Steinman, 2006). Each stage consisted of 24 practice trials, followed by 72 test trials. In the first stage (chocolate + I like), participants had to respond with a left-hand key (“*E*”) to chocolate and positive pictures and a right-hand key (“*I*”) to negative pictures. In the second stage (chocolate + I don’t like), participants had to respond with a left-hand key (“*E*”) to positive pictures and a right-hand key (“*I*”) to chocolate and negative pictures. The target stimuli were six pictures of chocolate. The attribute stimuli were six positive (IAPS#1610, IAPS#1750, IAPS#1920, IAPS#1999, IAPS#2057, IAPS#2209) and six negative pictures (IAPS#1275, IAPS#1301, IAPS#2900, IAPS#3300, IAPS#9470, IAPS#9561) from the International Affective Picture System (IAPS, Lang et al., 2008). During each trial, the target, or attribute, stimulus appeared in the center of the screen, and category reminder labels remained on the bottom. A SC-IAT score was computed for each participant using the *D*-measure with 600-ms error penalty (Greenwald et al., 2003). A more positive value indicated a more positive implicit attitude toward chocolate.

#### Chocolate Consumption

A so-called taste test was used to assess chocolate consumption. Participants were left alone for 8 min with 120 separately wrapped (5 g) Hershey chocolates of different flavors. Each participant was asked to taste and rate the chocolates on a 28-item questionnaire. After the time had expired, the chocolates were removed. Chocolate consumption was determined by weighing the chocolate before and after the task.

#### Behavioral Impulsivity

A response inhibition task (stop signal paradigm, Logan et al., 1997) was used to measure behavioral impulsivity. Studies have supported the reliability of this task (Congdon et al., 2012). A choice reaction time task was implemented, in which participants were instructed to respond as fast as possible to a visual stimulus, unless an auditory stop signal was presented after a variable delay. The stimuli for the response task were arrows presented in the center of the screen pointing left or right. Only the visual stimulus was presented during the no-signal trials (75% of the trials). Participants should have responded to the direction of the arrow with a left- (“q”) or right-hand key (“p”). The arrow was followed by an auditory stop signal (750 Hz, 75 ms) during the stop signal trials (25% of the trials), and participants should have withheld their responses. The stop signal delay was initially set to 250 ms and then adjusted dynamically depending on the response. If the participant successfully stopped, the delay was increased by 50 ms. If the participant failed to stop, the delay was decreased by 50 ms. The experiment consisted of a practice block of 32 trials and four test blocks of 64 trials. Stop signal reaction time (SSRT) was calculated by subtracting the mean stop signal delay from the mean no-signal reaction time. A higher SSRT indicates low response inhibition and high behavioral impulsivity. We assessed the reliability of this task by calculating SSRT separately for odd and even trials of each subject (Logan et al., 1997). The Pearson’s correlation coefficient between the two halves was 0.852.

#### Self-Reported Impulsivity

The 30-item Barratt Impulsiveness Scale Version 11 (BIS-11, Patton et al., 1995) was used to measure self-reported impulsivity. Participants rated how well each statement described them (e.g., “I do things without thinking”, “I buy things on impulse”) on a scale from 1 (Not at all) to 4 (very much). The means of all 30 items was calculated. Higher scores indicated more impulsiveness (Cronbach’s α = 0.825).

#### Data Analyses

Multiple regressions were used to test our predictions. The depletion condition was dummy-coded (0 = depletion, 1 = no-depletion). All continuous variables were standardized and interaction terms were computed from this score (Aiken and West, 1991). All regressions used z-standardized chocolate consumption as the dependent variable. *Post hoc* power analyses were conducted using G*Power 3.1 (Faul et al., 2009).

The first regression analysis was conducted to test the prediction that ego depletion and implicit preferences combine to influence food consumption. We entered the depletion condition and implicit preferences as predictors in the first step. We entered their two-way interaction in the second step. The power was 0.84, based on a sample size of 95 and an alpha of 0.05.

A second regression analysis was conducted to test the prediction that self-reported impulsivity, ego depletion, and implicit preferences combine to influence food consumption. We entered depletion condition, implicit preferences, and self-reported impulsivity as predictors in the first step. We entered all three two-way interactions between the predictors in the second step. We entered the three-way interaction in the third step. The power was 0.83, based on a sample size of 95 and an alpha of 0.05.

The third regression analysis was conducted to test the prediction that behavioral impulsivity, ego depletion, and implicit preferences combine to influence food consumption. We entered depletion condition, implicit preferences, and behavioral impulsivity as predictors in the first step. We entered all three two-way interactions between the predictors in the second step. We entered the three-way interaction in the third step. The power was 0.80, based on a sample size of 95 and an alpha of 0.05.

We also examined if, and how, trait impulsivity interacts with implicit preferences in predicting food consumption. Results are presented in the Supplemental Materials.

## Results

### Descriptive Statistics and Randomization Check

**Table 1** shows the descriptive statistics and zero-order correlations of the main variables measured in this study.

**Table 1:** Descriptive statistics and zero-order correlations of main variables.

	*M*	*SD*	2	3	4
1 Implicit chocolate preferences	0.21	0.35	0.097	-0.143	0.297^∗∗^
2 Self-reported impulsivity	2.22	0.31		0.041	0.142
3 Response inhibition (ms)	234.00	47.26			0.001
4 Chocolate consumption (g)	45.58	21.84			

*N = 95. Two-tailed Pearson’s correlations. ^∗∗^p < 0.01.*

No significant differences were found between the two experimental conditions in implicit preferences toward chocolate (*M* = 0.26, *SD* = 0.30 vs. *M* = 0.18, *SD* = 0.40, *t*(93) = –1.305, *p* = 0.195), self-reported impulsivity (*M* = 2.27, *SD* = 0.31 vs. *M* = 2.18, *SD* = 0.31, *t*(93) = –1.367, *p* = 0.175), or response inhibition (*M* = 231.67, *SD* = 50.06 vs. *M* = 236.39, *SD* = 44.63, *t*(93) = 0.484, *p* = 0.629). Therefore, the randomization of participants was successful.

### Manipulation Check and Mood

Participants in the depletion condition exerted more effort following the instructions for crossing out letters than participants in the no-depletion condition (*M* = 4.25, *SD* = 1.12 vs. *M* = 3.02, *SD* = 1.61, *t*(93) = –4.328, *p* < 0.001). Thus, the manipulation of ego depletion was successful. The depletion manipulation did not significantly influence positive (α = 0.770, *M* = 2.75, *SD* = 0.52 vs. *M* = 2.93, *SD* = 0.54, *t*(93) = 1.632, *p* = 0.106) or negative affect (α = 0.871, *M* = 1.72, *SD* = 0.56 vs. *M* = 1.64, *SD* = 0.52, *t*(93) = –0.664, *p* = 0.509).

### The Effects of Implicit Preferences and Depletion Condition on Chocolate Consumption

**FIGURE 1:**
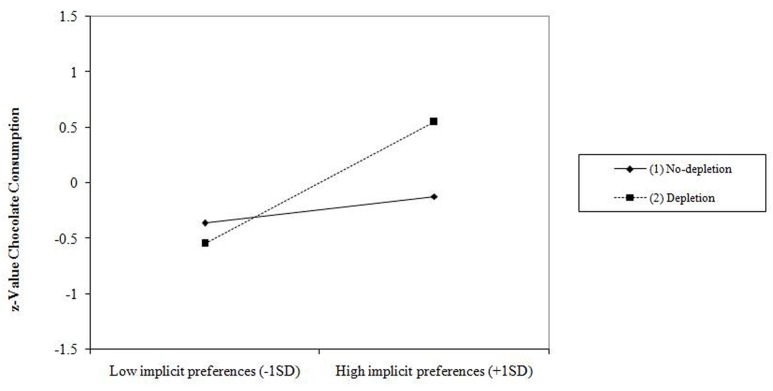
**Slopes for implicit preferences-chocolate consumption relationship across levels of ego depletion**.

Results are presented in **Table 2**. The regression analysis (*R*^2^ = 0.146) showed a significant main effect for implicit preferences (β = 0.548, *p* = 0.001) and a significant two-way interaction between implicit preferences and depletion condition (β = –0.337, *p* = 0.039). Simple slopes are plotted in **Figure 1**, which shows that implicit preferences positively predicted chocolate consumption in the depletion condition, β = 0.548, *p* = 0.001, but not in the no-depletion condition, β = 0.122, *p* = 0.322.

**Table 2:** Summary of multiple regression analysis for chocolate consumption with implicit preferences and depletion condition as predictors.

Predictor	Chocolate consumption: Step1						Chocolate consumption: Step2					
	*b*	*SE*	*t*	*p*	*LLCI*	*ULCI*	*b*	*SE*	*t*	*p*	*LLCI*	*ULCI*
Implicit preferences (*A*)	0.28	0.10	2.81	0.006	0.08	0.48	0.55	0.16	3.40	0.001	0.23	0.87
Depletion condition (*B*)	–0.26	0.20	–1.30	0.197	–0.65	0.14	–0.24	0.20	–1.25	0.215	–0.63	0.14
*A* ×*B*							–0.43	0.20	–2.10	0.039	–0.83	–0.02
*R*^2^	0.104						0.146					
*p* (*R*^2^)	0.006						0.002					
*ΔR*^2^	0.104						0.041					
*p* (*ΔR*^2^)	0.006						0.039					

*N = 95. LLCI = 95% confidence interval lower limit; ULCI = 95% confidence interval upper limit*.

### The Effects of Implicit Preferences, Depletion Condition, and Self-Reported Impulsivity on Chocolate Consumption

**FIGURE 2:**
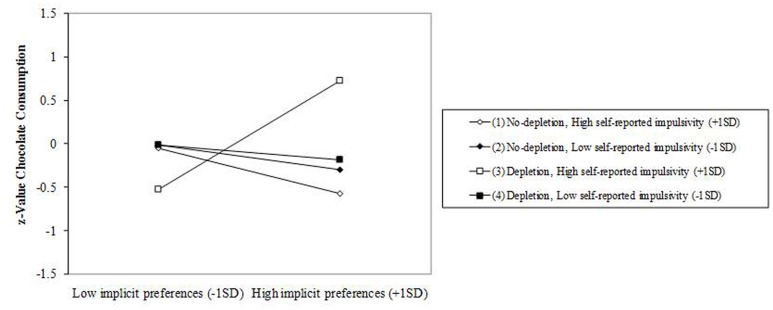
**Slopes for implicit preferences-chocolate consumption relationship across levels of ego depletion and self-reported impulsivity**.

Results are presented in **Table 3**. The regression analysis (*R*^2^ = 0.223) showed a significant main effect for implicit preferences (β = 0.566, *p* = 0.001), a significant two-way interaction between implicit preferences and depletion condition (β = –0.375, *p* = 0.029), and a significant two-way interaction between implicit preferences and self-reported impulsivity (β = 0.370, *p* = 0.012). These interactions were qualified by a significant three-way interaction (β = –0.324, *p* = 0.040). Probing the interaction (see **Figure 2**) revealed a single significant simple slope (β = 0.627, *p* = 0.007), reflecting that implicit preferences were positively associated with food consumption only among individuals high in self-reported impulsivity under the depletion condition.

**Table 3:** Summary of multiple regression analysis for chocolate consumption with implicit preferences, depletion condition, and self-reported impulsivity as predictors.

	Chocolate consumption: Step1						Chocolate consumption: Step2						Chocolate consumption: Step3					
Predictor	*b*	*SE*	*t*	*p*	*LLCI*	*ULCI*	*b*	*SE*	*t*	*p*	*LLCI*	*ULCI*	*b*	*SE*	*t*	*p*	*LLCI*	*ULCI*
Implicit preferences (*A*)	0.27	0.10	2.72	0.008	0.07	0.47	0.54	0.16	3.29	0.001	0.21	0.86	0.57	0.16	3.52	0.001	0.25	0.89
Depletion condition (*B*)	-0.23	0.20	-1.16	0.249	-0.63	0.17	-0.20	0.20	-1.00	0.322	-0.59	0.19	-0.17	0.19	-0.89	0.378	-0.55	0.21
Self-reported impulsivity (*C*)	0.10	0.10	0.99	0.233	-0.10	0.30	0.17	0.14	1.25	0.217	-0.10	0.45	0.15	0.14	1.12	0.264	-0.12	0.43
*A* ×*B*							-0.33	0.21	-1.61	0.111	-0.74	0.08	-0.47	0.21	-2.22	0.029	-0.90	-0.05
*A* ×*C*							0.15	0.10	1.51	0.135	-0.05	0.36	0.36	0.14	2.55	0.012	0.08	0.63
*B* ×*C*							-0.19	0.20	-0.97	0.335	-0.58	0.20	-0.18	0.19	-0.92	0.361	-0.56	0.21
*A* ×*B* ×*C*													-0.42	0.20	-2.08	0.040	-0.81	-0.02
*R*^2^	0.114						0.184						0.223					
*p* (R^2^)	0.011						0.006						0.002					
*ΔR*^2^	0.114						0.070						0.039					
*p* (ΔR^2^)	0.011						0.063						0.040					

*N = 95. LLCI = 95% confidence interval lower limit; ULCI = 95% confidence interval upper limit*.

### The Effects of Implicit Preferences, Depletion Condition, and Behavioral Impulsivity on Chocolate Consumption

**FIGURE 3:**
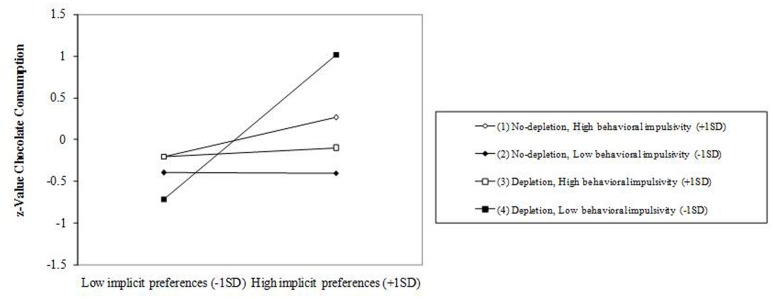
**Slopes for implicit preferences-chocolate consumption relationship across levels of ego depletion and behavioral impulsivity**.

Results are presented in **Table 4**. The regression analysis (*R*^2^ = 0.216) showed a significant main effect for implicit preferences (β = 0.458, *p* = 0.006), and a significant two-way interaction between implicit preferences and behavioral impulsivity (β = –0.355, *p* = 0.033). The interaction was qualified by a significant three-way interaction (β = 0.362, *p* = 0.029). Probing the interaction (see **Figure 3**) revealed a single significant simple slope (β = 0.865, *p* < 0.001), reflecting that implicit preferences were positively associated with food consumption only among individuals low in behavioral impulsivity under the depletion condition.

**Table 4:** Summary of multiple regression analysis for chocolate consumption with implicit preferences, depletion condition, and behavioral impulsivity as predictors.

Predictor	Chocolate consumption: Step1						Chocolate consumption: Step2						Chocolate consumption: Step3					
	*b*	*SE*	*t*	*p*	*LLCI*	*ULCI*	*b*	*SE*	*t*	*p*	*LLCI*	*ULCI*	*b*	*SE*	*t*	*p*	*LLCI*	*ULCI*
Implicit preferences (*A*)	0.29	0.10	2.84	0.006	0.09	0.49	0.52	0.17	3.16	0.002	0.19	0.85	0.46	0.16	2.80	0.006	0.13	0.78
Depletion condition (*B*)	-0.26	0.20	-1.31	0.194	-0.66	0.14	-0.25	0.20	-1.27	0.208	-0.64	0.14	-0.19	0.19	-0.96	0.341	-0.57	0.20
Behavioral impulsivity (*C*)	0.05	0.10	0.49	0.627	-0.15	0.25	-0.08	0.13	-0.62	0.539	-0.35	0.18	-0.15	0.13	-1.14	0.260	-0.42	0.12
*A* ×*B*							-0.36	0.21	-1.67	0.098	-0.78	0.07	-0.34	0.21	-1.65	0.102	-0.76	0.07
*A* ×*C*							-0.08	0.12	-0.67	0.505	-0.31	0.16	-0.41	0.19	-2.17	0.033	-0.78	-0.03
*B* ×*C*							0.30	0.20	1.48	0.141	-0.10	0.69	0.37	0.20	1.86	0.067	-0.03	0.76
*A* ×*B* ×*C*													0.53	0.24	2.21	0.029	0.05	1.00
*R*^2^	0.107						0.172						0.216					
*p* (*R*^2^)	0.016						0.009						0.003					
*ΔR*^2^	0.107						0.065						0.044					
*p* (*ΔR*^2^)	0.016						0.082						0.029					

*N = 95. LLCI = 95% confidence interval lower limit; ULCI = 95% confidence interval upper limit*.

## Discussion

The present study found that trait impulsivity and ego depletion interact to moderate the effect of implicit preferences on food consumption. In line with previous research, implicit preferences predicted the amount of food consumed among participants who exerted self-control and were depleted. Only participants high in self-reported impulsivity showed a ‘depletion and then eating according to impulses’ effect. This finding supported our hypothesis. However, the results on behavioral task differed. Participants low in behavioral impulsivity showed a consistent impulse-consumption effect under the depletion condition.

Few studies have measured impulsive precursors and explored situational or dispositional factors influencing impulsive and reflective processes within the dual-systems account of self-control (Milyavskaya et al., 2015). Researchers have proposed that measures of implicit preferences, atttentional bias, and approach-avoidance bias are valid impulse strength proxies (Hofmann et al., 2009b; Sheeran et al., 2013). Our study confirmed the predictive validities of implicit attitude measures (IAT) under a depletion condition, which supported previous findings (Hofmann et al., 2007; Friese et al., 2008b). These results support the dual-systems perspective of impulse and self-control. The dual-systems model predicts that the relative influence of impulsive processes increases under low self-control strength (Hofmann et al., 2009b). We extended previous work by examining the moderating role of trait impulsivity in the ‘eating on impulse after depletion’ effect. We tried to conjoin variables that shift the relative impact of impulsive and reflective precursors on behavior, and explored how they interact. Investigating the potential moderating influences of individual characteristics in the ego depletion effect may also reveal boundary conditions and underlying processes (Hagger et al., 2010). Self-report questionnaires may measure a different aspect of impulsivity than behavioral tasks. Therefore, we used both methods to measure trait impulsivity. Furthermore, the dependent variable and trait measures were collected at two different time points, thus, reducing the chance of a common method bias (Podsakoff et al., 2003). However, the sample size warrants that results about the three-way interactions be regarded as exploratory. The current results suggest that the combination of dispositional (i.e., high self-reported impulsivity) and situational (i.e., ego depletion) variables might render people vulnerable to impulsive behavior. Nevertheless, results differed when trait impulsivity was measured with a behavioral paradigm (i.e., stop signal task).

Researchers agree that impulsivity is not a unitary construct (Reynolds et al., 2006). Researchers recently performed a comprehensive latent-variable examination of the facets underlying impulsive behavior, and their relations with self-reported impulsivity. Results showed that behavioral impulsivity was unrelated to self-reported impulsivity (Stahl et al., 2014). The correlation between self-reported impulsivity (measured with BIS-11) and behavioral impulsivity (i.e., inefficient response inhibition in a stop signal task) was weak in our study. This result was similar to another study using an all-female sample (Guerrieri et al., 2007b). Furthermore, individuals with high self-reported impulsivity or low behavioral impulsivity were more vulnerable to temptation when depleted. These results support previous research showing that impulsivity is a multidimensional construct (e.g., Reynolds et al., 2006; Stahl et al., 2014; Bongers et al., 2015). Self-reported impulsivity, but not behavioral impulsivity, was associated with an attentional bias for unhealthy foods in people with obesity (Bongers et al., 2015). We discussed the interactions between self-reported and behavioral impulsivity separately because of their divergence.

The Barratt Impulsiveness Scale is a widely used generic measure of impulsiveness that focuses on low self-control (Patton et al., 1995). Impulsivity and self-control were once thought to represent two end points of the same dimension (Duckworth and Kern, 2011). Some researchers believed these variables predicted a variety of adaptive behaviors (de Ridder et al., 2012). The present study found that participants with low self-reported impulsivity were more resistant to the effects of ego depletion on eating behavior. In contrast, participants with high self-reported impulsivity were more vulnerable to the effect of ego depletion on impulsive eating. Our results parallel previous findings showing that trait self-control served as a buffer against the influence of depletion on self-control (Muraven et al., 2005; Wang et al., 2015). One reason for this finding is that dispositional self-control reflects self-control reserves (Muraven et al., 2005). For example, the strength model proposes that people with high dispositional self-control (i.e., low impulsivity) have more cognitive resources to impede the depletion effect (Baumeister et al., 2006). Another reason for this finding is that depleted individuals who have less trait-level resources try to conserve their remaining resources and exhibit larger depletion effects (Buczny et al., 2015). Future studies are needed to test these possibilities.

Behavioral measures operationalize impulsivity as a decreased prepotent response inhibition (Logan et al., 1997). The current study examined if, and how, response inhibition moderated the depletion effect. Contrary to our hypothesis, results suggested that individuals with effective response inhibition (i.e., low behavioral impulsivity) are more vulnerable to the effect of depletion. No known previous studies have explored the effect of response inhibition on depletion susceptibility. The small sample size and single study nature of our manuscript warrant future research. The assumption that response inhibition increases the susceptibility to self-control failure is premature. We found just two relevant studies offering possible explanations for our findings. One found that higher fluid intelligence was associated with greater depletion (Shamosh and Gray, 2007). Authors proposed that cognitive abilities influence the tendency to consume self-control resources during the first task. Another study showed that high involvement and good self-control facilitate performance in the first task, but may jeopardize performance in a subsequent unexpected task (Ein-Gar and Steinhart, 2011). Researchers have termed this phenomenon the “sprinter effect.” Both propositions assume that some individuals exert more effort in the initial self-control task and are more susceptible to the depletion effect. Future research needs to examine this assumption.

Our research has implications for the study of ego depletion. The lack of a main effect of ego depletion on food consumption is consistent with recent research (Carter et al., 2015). Some researchers have contended that the ego depletion effect is substantially smaller than published literature implies (Carter and McCullough, 2013, 2014; Tuk et al., 2015; see Hagger and Chatzisarantis, 2014, for a response to this argument). Some researchers have questioned the authenticity of the depletion effect (Carter et al., 2015). One pre-registered replication study found no evidence of an ego depletion effect (Lurquin et al., 2016). Similarly, a multi-lab pre-registered replication study found minimal evidence of a depletion effect (Hagger et al., 2016; for a commentary, see Baumeister and Vohs, 2016). Our research provides a plausible explanation for these controversies in the food domain. Variances in impulses exist. Ego depletion and increased reliance on impulses may lead to higher food consumption for some, and decreased consumption for others. The main effect of ego depletion on consumption only emerges in a sample of individuals who have higher mean impulses toward a particular food (Friese and Hofmann, 2009). Our results suggest that individuals differ in their vulnerability to the depletion effect. Thus, ego depletion may not be equally likely in all people. Rather, this effect depends on individual difference variables in depletion sensitivity (Salmon et al., 2014), trait self-control (Imhoff et al., 2014), and trait impulsivity. Future studies need to systematically examine factors that moderate the ego depletion effect. Such studies may provide insight into the mechanisms underlying the depletion effect and ways to attenuate or overcome this effect.

The present study had several limitations that warrant consideration when interpreting the results. First, we only used one task to manipulate depletion and examined just food consumption behavior as an outcome. Future studies need to examine whether the current findings extend to other types of depletion manipulation and self-control outcomes. Second, participants were limited to female undergraduate students and the sample size was too small to accurately interpret three-way interactions. Future research is needed using larger and more representative samples to improve the generalizability of the present findings. Third, we did not measure participants’ dietary restraint standards, which the dual-systems theory of self-control proposes might serve as reflective precursors (Hofmann et al., 2009b). The effect of depletion on impulsive eating may be more pronounced among individuals who are strongly motivated to refrain from high-calorie foods. Future research is needed to explore this possibility.

## Conclusion

The present study replicated the finding that ego depletion leads to eating according to implicit preferences. Furthermore, individual differences in impulsivity were associated with vulnerability to depletion. These results have implications for the dual-systems theory of self-control and ego depletion studies. More work is needed to examine whether the current findings apply to various self-control domains.

## Author Contributions

YW conceived and designed the study. YW, JZ, and YH drafted the paper; YW, JZ, YF, GW, XC, and LW performed the experiments; YW, YH, JZ, and YF analyzed the data.

## Conflict of Interest Statement

The authors declare that the research was conducted in the absence of any commercial or financial relationships that could be construed as a potential conflict of interest. The reviewer JC and the handling Editor declared their shared affiliation, and the handling Editor states that the process nevertheless met the standards of a fair and objective review.
